# The Book World of Medicine and Science

**Published:** 1899-07-08

**Authors:** 


### The Book World of Medicine and Science.

The Natural Mineral Waters of Harrogate. By F. William Smith, M.D. (London: Dawbarn and Ward, Limited. 1899. Pp. 101. Price 1s.)

Of the making of books there is no end. And no books are more obviously "made" than those in which a physician extols the merits of the watering place in which he happens to reside. Of these books the jauntily-written and rather spasmodic little work now before us is an excellent sample. It happens to deal with Harrogate. But for the maps and analyses it might stand equally well for Bath, Buxton, Leamington, or Matlock, for it describes the peculiarly low death-rate, the highly satisfactory meteorological conditions, and the undoubted superiority over all other watering places, of the town it deals with. Having said so much one is glad to have an opportunity of calling attention to the very real benefits to be obtained from treatment at our British watering places. Unhappily the latter-day Naaman is only too ready to disparage "Abana and Pharpar, rivers of Damascus." He prefers to be fleeced, in dubious company, in insanitary German hotels. Amongst our British watering places Harrogate, no doubt, occupies a very high position, and Dr. Smith is certainly justified in printing in large letters the exultant statement that in Harrogate is the strongest sulphur well in Europe. And, as Dr. Smith points out, the sulphur springs of Harrogate are unrivalled in the treatment of "bilious" dyspepsia, while the iron springs are of great value in cases of anæmia and chlorosis.

A Century of Vaccination. By W. S. Tebb, M.A., M.D. (London: Swan Sonnenschein and Co., Limited. 1899. Pp. 452. Price 6s. Second Edition.)

We have so recently criticised this work that the appearance of a second edition calls for little comment, and only induces some very common-place reflections on human credulity and wrong-headedness. No doubt Dr. Tebb has some grounds for the jubilant tone that he adopts in his second preface. But surely a sense of humour should have prevented him from remarking that the researches of Dr. Creighton and Professor Crookshank—which have, he laments, been "so generally ignored by the profession"—have conclusively proved that cow-pox is a disease radically different from that against which it is said to protect. Even if these gentlemen had proved their point, the power of cow-pox to protect would not be shaken. But, since Dr. Creighton believes cow-pox to be a modified syphilis, and Professor Crookshank does not, their "conclusive proofs" are mutually destructive.

The Diseases of Children: A Clinical Handbook. By George Elder, M.D., F.R.C.P.Ed., and J. S. Fowler, M.B., F.R.C.P.Ed. (London: Charles Griffin and Co. 1899. Price 10s. 6d.)

This is a book which will be found useful both to read and to refer to; and useful also both to the student and the practitioner—to the former because it gives in comparatively small compass a large amount of information about children's diseases, and to the latter because by its constant insistence on matters pertaining to diagnosis it refreshes the mind on the point in regard to which the general practitioner, in the hurry of his daily work, tends most to go astray. The book is well arranged, the balance between the various subjects dealt with being properly maintained, and all throughout one feels that its authors are men accustomed to teach as well as merely to practice their profession. The first section is devoted to anatomy, physiology, feeding in infancy and childhood and to general therapeutics, subjects in regard to all of which the infant and the child differ so widely from the adult as to render a special study of their peculiarities quite essential. Then comes a long section on diagnosis, which really is the key to the book, as indeed it is to the successful management of disease. This section is very good and well up to date, more so perhaps than some of the chapters dealing with the several diseases. For example, in the paragraph on lumbar puncture in the diagnosis of spinal disorders we find a reference to the diplococcus of Weichselbaum as one of the organisms that may be met with, whereas in the article on cerebro-spinal meningitis no mention is made of this organism as a cause of the disease. Section III. treats of the various diseases to which children are especially liable, or which in children present peculiarities separating them from the same diseases in adults, and although it would seem that this portion of the work is kept to its present moderate dimensions by omitting a good deal of pathological detail, to the practitioner this is perhaps an advantage, whlle to the student it is no disadvantage if he recognises the true position which a book of this sort ought to hold as a handbook not of medicine but of that side of medicine which is special to children, a knowledge of the general subject being presupposed. With this limitation we can speak in terms of the highest praise of this which forms the main part of the book. As in every book there are little points here and there to which exception may be taken, and, of course, a small work can never be a complete treatise on the subject, but as a guide to the clinical study of children's diseases the book before us is very satisfactory, and an immensity of well-arranged information has been got into its less than 400 pages. Finally, a word of praise must be given to the plates, which are not only very good in themselves but are well selected and highly characteristic of the points they serve to illustrate.

### NOTES ON BOOKS.

The Great Eastern Railway Company have issued a little pamphlet by Percy Lindley in praise of the plan of taking one's holidays in the old Flemish cities and in the Ardennes. For a short holiday nothing could be more pleasant, and this *brochure*, with its pretty illustrations, well portrays the sights which are available even for tourists whose time is short.

